# Prevalence and factors associated with possible cases of familial hypercholesterolemia in Brazilian adults: a cross-sectional study

**DOI:** 10.1038/s41598-023-47692-7

**Published:** 2023-11-22

**Authors:** Ana Carolina Micheletti Gomide Nogueira de Sá, Crizian Saar Gomes, Elton Junio Sady Prates, Luisa Campos Caldeira Brant, Deborah Carvalho Malta

**Affiliations:** 1 Postgraduate Nursing Program, Nursing School, Federal University of Minas Gerais (UFMG), Avenue Alfredo Balena, No 190, Santa Efigênia, Belo Horizonte, MG CEP 30130-100 Brazil; 2 Postgraduate Program in Public Health, Faculty of Medicine of the Federal University of Minas Gerais (UFMG), Belo Horizonte, MG Brazil; 3 Postgraduate Nursing Program, Nursing School, Federal University of Minas Gerais (UFMG), Belo Horizonte, MG Brazil; 4 Faculty of Medicine, Federal University of Minas Gerais (UFMG), Belo Horizonte, MG Brazil; 5 Department of Maternal and Child Nursing and Public Health, School of Nursing, Federal University of Minas Gerais (UFMG), Belo Horizonte, MG Brazil

**Keywords:** Cardiology, Diseases

## Abstract

This study aimed to estimate the prevalence of possible cases of FH and analyze associated factors in the adult Brazilian population. Cross-sectional study with laboratory data from the Brazilian National Health Survey, with 8521 participants. Possible cases of FH were defined according to the Dutch Lipid Clinic Network criteria. The prevalence and 95% confidence intervals (95% CI) of possible cases of FH were estimated according to sociodemographic variables, lifestyle, diabetes, hypertension, altered tests, treatment and self-rated health. Logistic regression was used to analyze the associations. The prevalence of possible cases of FH was 0.96%, higher in women, between 45 and 59 years, white race/skin color and others, less education, people with diabetes, hypertension and total cholesterol ≥ 310 mg/dL. The presence of FH was positively associated with regular self-rated health (OR 1.96; 95% CI 0.99–3.84), poor/very poor (OR 3.02; 95% CI 1.30–7.03) and negatively with black race/skin color (OR 0.10; 95% CI 0.02–0.46) and complete elementary school, incomplete high school (OR 0.47; 95% CI 0.23–0.98) and complete high school and more (OR 0.45; 95% CI 0.21–0.95). FH affects 1:104 Brazilian adults, these findings contribute to understanding the burden of disease in Brazil. Due to the scarcity of studies on FH in low- and middle-income countries, further studies are desirable.

## Introduction

Familial hypercholesterolemia (FH) is a genetic disorder of lipoprotein metabolism. The occurrence of FH is due to mutations in the gene of low-density lipoprotein (LDLR), alloprotein B (APOB) or proprotein convertase subtilisin/kexin type 9 (PCSK9) gene^1,2^. Approximately 95% of FH cases are due to LDLR mutations, with decreased or lost function^3,4^. Considered severe due to the increased risk of premature coronary artery disease (CAD), accounting responsible for 5–10% of cardiovascular events under the age of 50^2^.

The prevalence in the world population varies between 1/200–250^3^ and 1/500^1^, corresponding to 14–34 million cases^3,5^. In Brazil, data from the Longitudinal Study of Adult Health (ELSA-Brazil) estimated that the prevalence of FH was 1 in 263^6^. Diagnosis is based on low-density lipoprotein cholesterol (LDL-Cholesterol) values above 190 mg/dL for adults^3,7^ and on family history of early CAD^7^. Homozygous FH (HoFH) is rarer (world-wide prevalence between 1/160,000 and 1/320,000) and can cause cardiovascular involvement from childhood, and many individuals develop CAD and die before the age of 30 LDL-Cholesterol levels are above 500 mg/dL, possibly leading to the onset of cholesterol deposits in the tendons, skin and vascular tissues^7^.

The World Health Organization (WHO) emphasizes that FH is a public health problem and meets the criteria for identifying population-based diseases for early diagnosis and treatment^5,8^, aiming to reduce cardiovascular mortality in the general population. The identification can be made by analyzing the lipid profile, determining total cholesterol (TC) and LDL-cholesterol^3,7,8^. Despite all the negative repercussions of FH, the disease is globally underdiagnosed and undertreated^8,9^, amplifying the burden of cardiovascular disease (CVD) in low- and middle-income countries^2,5^. The identification of FH is a challenge^7^ because many countries do not have records with these observations^10^.

In this sense, some criteria have been proposed to standardize FH diagnosis, such as the Dutch Lipid Clinic Network (DLCN) clinical score^1,3,7,8^. The Brazilian FH Directive^1^ recommends using the DLCN for simplicity of application, although a validation for the Brazilian population is not yet available.

In Brazil, there is little information on the population diagnosis of FH^5^. Some studies describe age, race, overweight and obesity as factors associated with FH^6,11^, but studies in the country that identify are scarce. This study advances by identifying, through laboratory tests of the Brazilian National Health Survey (PNS—*Pesquisa Nacional de Saúde*), the most extensive health survey in Brazil^12^, possible cases of FH in adults. Considering the importance of early determination of FH to achieve a reduction in morbidity and mortality through guidelines and necessary therapeutic measures^13^, possible cases of FH in adults. It is worth noting that investigating the factors associated with FH will contribute to a better understanding of the determinants of this phenomenon among adults in the country, which can be useful for designing strategies to address FH in Brazil, aiming to reduce the burden of FH through necessary guidance and therapeutic measures^13^.

In this context, the aim of this study was to estimate the prevalence of possible cases of FH and analyze the associated factors in the adult Brazilian population.

## Material and methods

### Study design

This is a cross-sectional study with data from laboratory tests of PNS between 2014 and 2015. The PNS is a nationwide, household-based survey conducted by the Brazilian Institute of Geography and Statistics in partnership with the Ministry of Health^14^.

### Sample, eligibility criteria and data collection

The PNS used a three-stage probabilistic sample and obtained records from 64,348 households and 60,202 adult respondents. To carry out the laboratory tests, a sub-sample collection of 25% of the census sectors surveyed was planned^12,14^.

In this study, the PNS subsample of 8,952 people was used. Adults under 20 years old with LDL-cholesterol below 150 mg/dL were excluded, due to the diagnostic possibility of FH being in adults from 20 years of age onwards years with levels above 190 mg/dL^1^. For the eligibility of possible FH cases, the DLCN score was adopted as proposed in the Brazilian FH Guideline^1^ and WHO^8^. Participants with unsuitable blood samples for analysis were also excluded.

The study adopted post-stratification weights according to sex, age, education and region, aiming to establish estimates for the Brazilian adult population^12^.

Peripheral blood collections were performed at any time of day^15^ following the protocol that does not require fasting for cholesterol measurement^7^. TC, LDL-Cholesterol and high density lipoprotein (HDL-Cholesterol) were collected in tubes with gel. The clot was retracted for 30 min and after centrifugation was performed and the samples were sent under refrigeration at 2 to 8ºC, with temperature control in the steps. These parameters were measured by an automated enzymatic/colorimetric method^15^. Further methodological details are available in other publications^12,14,15^.

### FH definition

FH was defined according to the diagnostic criteria of the DLCN^1,8^ adapted^16^, using the information available in the PNS database and based on population studies^6,11,17,18^.

The DLCN score classifies FH into three categories: certainty above 8 points, probable 6 to 8 points and possible 3 to 5 points, according to the diagnostic criteria below:

- Family history: first-degree relative with premature coronary/vascular disease (male < 55 years and woman < 60 years) or adult first- or second-degree relative with TC > 290 mg/dL (1 point); first-degree relative with tendon xanthoma and/or corneal arch or first-degree relative 260 mg/dL (2 points);
- Clinical history: personal history of premature CAD (2 points); and/or premature cerebrovascular disease (1 point). Considering as premature, under 55 years old for men and under 60 years old for women;
- Physical examination: xanthoma (6 points); corneal arch under 45 years old (4 points);
- LDL-Cholesterol Levels (mg/dL): 155–189 (1 point), 190–249 (3 points), 250–329 (5 points), ≥ 330 (8 points);
- DNA analysis (8 points).

Due to the variables collected in the PNS, this study used the diagnostic criteria of the DLCN score referring to LDL-cholesterol levels measured in laboratory tests and history of premature CAD and/or stroke assessed by self-reported diagnosis in the PNS questionnaire, which allowed estimating only possible cases of FH.

Therefore, possible cases of FH were defined using the following DLCN score criteria:

#### Criterion 1 (laboratory)

Assessed by LDL-Cholesterol ranges only. A dichotomous analysis was performed with or without FH, calculated by LDL-Cholesterol levels (mg/dL): 155–189 (1 point); 190–249 (3 points); 250–329 (5 points); ≥ 330 (8 points). Possible cases of FH were considered reaching the cutoff point from 3 to 5 points in the score and people who reached below 3 points in the score were considered without FH.

#### Criterion 2 (laboratory plus premature CAD and/or stroke)

Assessed by LDL-cholesterol ranges and self-report of premature CAD and/or stroke. The dichotomous analysis was performed with or without FH by the cut-off point of 3 to 5 points, calculated by: LDL-Cholesterol levels (mg/dL): 155–189 (1 point), 190–249 (3 points), 250–329 (5 points) and ≥ 330 (8 points); Premature CAD (2 points) and/or premature stroke (1 point), in male under 55 years and in female under 60 years. Possible cases of FH were considered when reaching 3 to 5 points in the score.

### Variables

This study included variables related to possible cases of FH, sociodemographic, lifestyle, additional risk factors for CVD, altered laboratory tests, treatment and health self-assessment. To construct the variables, questionnaires on lifestyles and chronic diseases and laboratory tests measured by the PNS were used. The questions used in the construction of the variables are shown in Supplementary  Table 1.

### Outcome variable

For analysis of associated factors, possible cases of FH defined by criterion 2 were used as the outcome variable.

### Explanatory variables

#### Sociodemographic

Gender (male and female); age (≥ 20 years old); Education (illiterate and incomplete elementary school, complete elementary school and incomplete high school, complete high school and more); Race/skin color (white and others that corresponded to yellow and indigenous; black; brown); Regions of Brazil (North, Northeast, Southeast, South and Center-West).

#### Lifestyle

Overweight or obesity: classified by body mass index (BMI), as normal/underweight (BMI < 25 kg/m^2^), overweight (BMI between 25 to 29 kg/m^2^) and obesity (BMI ≥ 30 kg/m^2^)^19^. Calculated by measured weight and height; Sufficient physical activity (PA) in free time (yes; no): the practice of 150 min per week of PA of light or moderate intensity, or at least 75 min per week of vigorous intensity, was considered active, regardless of the number of days of practice per week^20^; Consumption of red meat with fat (yes; no); Binge drinking (yes; no): defined by the concept of “binge drinking”^21^ (minimum consumption of 4 doses for women and 5 doses for men on a single occasion); Smoking (yes; no): positive responses to the use of tobacco products were considered as smokers.

#### Health self-assessment

Categorized as very good/good, regular and very poor/poor.

### Other variables included in the study

#### Additional risk factors for CVD

Diabetes (yes; no): glycated hemoglobin values ≥ 6.5%^22^ and self-reported diagnosis were considered; Hypertension (yes; no): Self-reported diagnosis and blood pressure measurements were used, considering values ≥ 140/90 mmHg^23^.

#### Altered laboratory tests

TC ≥ 310 mg/dL (yes; no); HDL-Cholesterol ≤ 40 mg/dL (yes; no).

#### Treatment report

Report of antihypertensive treatment (yes; no); Lipid-lowering treatment report (yes; no).

### Statistical analyses

Prevalence was estimated as proportions (%) and 95% confidence intervals (95% CI) for LDL-Cholesterol levels based on the DLCN considering sociodemographic characteristics; and for possible cases of FH according to criteria 1 and 2 according to sociodemographic characteristics, additional risk factors for CVD, altered laboratory tests, treatment report, lifestyle and health self-assessment. Bivariate analyzes were performed using the chi-square test, with a significance level of 5% (*p* ≤ 0.05).

To analyze the associations between the outcome and explanatory variables, the logistic regression model was applied and the Odds Ratio (OR) and 95% CI were calculated. For the bivariate analyses, the crude ORs (cOR) were estimated. In the multivariate analysis, the variables that presented a *p* value < 0.20 were included in the bivariate analyzes and the adjusted ORs (aOR) were estimated. In the final model, the variables that presented a *p* value ≤ 0.05 were considered as associated factors.

For data processing and analysis, the Software for Statistics and Data Science (Stata) version 14 was used, using the survey module that considers complex sample designs and unequal selection probabilities, applying post-stratification weights in all analyzes.

The dataset is available in the PNS repository (www.pns.fiocruz.br). The PNS was approved by National Committee of Ethics in Research, Ministry of Health, under Opinion 328,159. Participation was voluntary and the confidentiality of information guaranteed.

## Results

The sub-sample of PNS laboratory consisted of 8952 people. There was a loss of 418 samples (insufficient material, hemolysis, sample loss). Adults under 20 years old with LDL-Cholesterol below 150 mg/dL were excluded (n = 13). Thus, the sample consisted of 8,521 participants remained at this stage. Following the application of the definitions of possible cases of FH by the criteria adopted in this study, the sample for criterion 1 the sample covered 66 adults and for criterion 2, it corresponded to 87 adults.

The prevalence of LDL-Cholesterol by range DLCN, for levels (mg/dL) between: 155–189 was 4.84%; 190–249 was 0.64%; and 250–329 was 0.044%. Nine people with LDL-Cholesterol levels between 155 and 189 had premature CAD and one person with levels between 190 and 249 had premature stroke (data not shown) (Supplementary Table 2).

The prevalence of possible cases of FH according to criterion 1 was 0.69%, 0.89% in women, 0.91% in white race/color and others, 1.13% in those aged 60 years and over and 1.08% in the least educated. The prevalence of possible cases of FH by criterion 2 was 0.96%, 1.22% in women, 1.74% between 45 and 59 years old, 1.13% in white race/color and others and 1.55% in the less educated (Table 1).

**Table 1:** Prevalence of possible cases of FH and 95% CI in adults by criteria according to sociodemographic characteristics, Brazilian National Health Survey, Brazil, 2014–2015.

Variables	n	Possible cases of FH by criterion 1			Possible cases of FH by criterion 2		
		(3–5 points)			(3–5 points)		
		(n = 66)			(n = 87)		
		%	95% CI	*p*	%	95% CI	*p*
Total	8521	0.69	0.50–0.94		0.96	0.73–1.26	
Sex	8521						
Male		0.46	0.27–0.78	0.043	0.67	0.41–1.08	0.043
Female		0.89	0.60–1.32		1.22	0.87–1.70	
Age							
20–29		0.47	0.15–1.45	0.044	0.49	0.16–1.45	0.004
30–44		0.32	0.18–0.56		0.49	0.24–0.97	
45–59		0.92	0.53–1.60		1.74	1.17–2.57	
60 years and older		1.13	0.70–1.85		1.13	0.69–185	
Race/skin color	8521						
White and others		0.91	0.60–1.40	0.012	1.13	0.77–1.66	0.055
Black		0.052	0.007–0.37		0.14	0.03–0.61	
Brown		0.56	0.36–0.88		0.94	0.62–1.26	
Region	8521						
North		0.68	0.40–1.15	0.771	0.86	0.54–1.35	0.714
Northeast		0.64	0.42–0.97		0.83	0.57–1.20	
Southeast		0.69	0.37–1.27		0.92	0.54–1.55	
South		0.60	0.28–1.28		1.224	0.63–2.37	
Center-West		1.05	0.57–1.92		1.21	0.69–2.11	
Education	8521						
Illiterate/Incomplete elementary school		1.08	0.74–1.57	0.019	1.55	1.10–2.17	0.002
Complete elementary school/incomplete high school		0.46	0.22–0.95		0.62	0.34–1.13	
Complete high school and more		0.43	0.21–0.88		0.58	0.31–1.06	

The category not having FH was used to calculate the prevalence, but it is not shown in the table. FH: familial hypercholesterolemia. 95% CI: 95% confidence interval.

The prevalence of possible cases according to criterion 1 was higher in people with diabetes (1.33%) and TC ≥ 310 mg/dL (59.95%). According to criterion 2, the prevalence of possible cases of FH was higher in people with hypertension (1.75%), diabetes (1.85%) and TC ≥ 310 mg/dl (59.95%), 3.29% reported taking lipid-lowering treatment and 1.62% anti-hypertensive treatment, however, without statistically significant differences (*p* > 0.05) (Supplementary Table 3). In supplementary Table 4, the prevalence of possible cases according to criterion 1 is presented, according to selected variables (sociodemographic, lifestyle and health self-assessment).

By criterion 2, in the bivariate analysis, being female (cOR 1.84; 95% CI 1.01–3.36), aged between 45 and 59 years (cOR 3.59; 95% CI 1.12–11.48) and health self -regular assessment (cOR 2.31; 95% CI 1.25–4.30) and poor/very poor (cOR 3.94; 95% CI 1.79–8.70) were positively associated with FH. Being of black race/color (cOR 0.13; 95% CI 0.03–0.56), complete elementary school and incomplete high school (cOR 0.39; 95% CI 0.20–0.79), complete elementary school and incomplete high school (cOR 0.39; 95% CI 0.20–0.79) or complete high school and more (cOR 0.37; 95% CI 0.18–0.75) and practice PA (cOR 0.42; 95% CI 0.17–0.84) were negatively associated with FH (Table 2).

**Table 2:** Prevalence and factors associated with possible cases of FH by criterion 2 in adults according to variables selected, Brazilian National Health Survey, Brazil, 2014–2015.

Variables	n*	Possible cases of FH by Criterion 2 (n = 87)				
		%	95% CI	cOR	95% CI	p**
Total	8521	0.96	0.73–1.26			
Sex	8521					
Male		0.67	0.41–1.08	1		0.046
Female		1.22	0.87–1.70	1.84	1.01–3.36	
Age	8521					
20–29		0.49	0.16–1.45	1		
30–44		0.49	0.24–0.97	0.99	0.27–3.63	0.99
45–59		1.74	1.17–2.57	3.59	1.12–11.48	0.031
60 years and older		1.13	0.69–185	2.32	0.70–7.71	0.17
Race/skin color	8521					
White and others		1.13	0.77–1.66	1		
Black		0.14	0.03–0.61	0.13	0.03–0.56	0.007
Brown		0.94	0.62–1.26	0.83	0.47–1.45	0.507
Region	8521					
North		0.86	0.54–1.35	1		
Northeast		0.83	0.57–1.20	0.97	0.53–1.76	0.913
Southeast		0.92	0.54–1.55	1.07	0.53–2.17	0.842
South		1.224	0.63–2.37	1.43	0.63–3.24	0.385
Center-West		1.21	0.69–2.11	1.42	0.68–2.95	0.35
Education	8521					
Illiterate/Incomplete elementary school		1.55	1.10–2.17	1		
Complete elementary school/incomplete high school		0.62	0.34–1.13	0.39	0.20–0.79	0.009
Complete high school and more		0.58	0.31–1.06	0.37	0.18–0.75	0.006
Body Mass Index	8429					
Low/normal		0.74	0.44–1.23	1		
Overweight		1.19	0.79–1.78	1.62	0.84–3.12	0.153
Obesity		0.97	0.53–1.77	1.32	0.59–2.92	0.500
Physical activity	8511					
No		1.11	0.82–1.49	1		0.016
Yes		0.42	0.20–0.88	0.38	0.17–0.84	
Consumption of red meat with fat	8041					
No		1.08	0.79–1.48	1		0.373
Yes		0.79	0.43–1.46	0.73	0.37–1.46	
Binge drinking	8521					
No		0.97	0.72–1.29	1		0.900
Yes		0.91	0.37–2.20	0.94	0.37–2.42	
Smoking	8514					
No		0.87	0.64–1.18	1		0.156
Yes		1.45	0.77–2.17	1.67	0.82–3.40	
Health self-assessment	8514					
Very good/good		0.62	0.40–0.97	1		
Regular		1.42	0.93–2.16	2.31	1.25–4.30	0.008
Very poor/poor		2.40	1.26–4.50	3.94	1.79–8.70	0.001

*Missing data not presented. The category not having FH was used to calculate the prevalence, but it is not shown in the table. FH: familial hypercholesterolemia. 95% CI: 95% confidence interval. cOR: Crude Odds Ratio. **p value of cOR.

By criterion 2, in the multivariate analysis, regular health self-assessment (aOR 1.96; 95% CI 0.99–3.84) and poor/very poor (aOR 3.02; 95% CI 1.30–7.03) were associated with if positively to FH. On the other hand, having completed elementary school and incomplete high school (aOR 0.47; 95% CI 0.23–0.98) or having completed high school and more (aOR 0.45; 95% CI 0.21–0.95) and being of black race/color (aOR 0.10; 95% CI 0.02–0.46) were negatively associated with FH (Table 3).

**Table 3:** Factors associated with possible cases of FH by criterion 2 in Brazilian adults, Brazilian National Health Survey, Brazil, 2014–2015.

Variable	aOR	95% CI	p**
Education			
Illiterate/Incomplete elementary school	1		
Complete elementary school/incomplete high school	0.47	0.23–0.98	0.044
Complete high school and more	0.45	0.21–0.95	0.036
Race/skin color			
White and others	1		
Black	0.10	0.02–0.46	0.003
Brown	0.72	0.42–1.23	0.228
Health self-assessment			
Very good/good	1		
Regular	1.96	0.99–3.84	0.050
Very poor/poor	3.02	1.30–7.03	0.010

FH: familial hypercholesterolemia. 95% CI: 95% confidence interval. aOR: Adjusted Odds Ratio (Final Model). **P-value of aOR.

## Discussion

This study identified the prevalence of possible cases of FH and associated factors in a representative sample of Brazilian adults. The frequency of FH in adults in Brazil was 1 case in 104 individuals, affecting more women, those aged 45–59 years, less educated, with TC ≥ 310 mg/dL, hypertension and diabetes. Regular and poor/very poor self-rated health were positively associated with possible cases of FH, while higher education and black race/color were negatively associated. The early identification of individuals with FH is relevant as it can enable early treatment with statins, capable of reducing cardiovascular events^24,25^ in these individuals by up to 76%^25^.

In this study, the prevalence of possible cases of FH was higher than that estimated in a meta-analysis that found 0.40% (frequency of 1:250)^10^ and in relation to the Elsa-Brazil study that identified 0.40% (1:263)^6^. Studies have identified the following prevalence of FH in adults: 0.40% in the United States (1:250)^17^, 0.30% in China (1:256)^11^, 0.85% in France (1:120)^18^ and 0.73% in Denmark (1:137)^26^, and the frequency in this study approached these last two countries^18,26^. Regarding the diagnostic criteria adopted in these studies, all of them used adapted versions due to the unavailability of information in the databases used, such as the identification of xanthomas through physical examination^11,17,18,26^, genetic testing^11,17,18^ or family history^17^. Furthermore, it is important to highlight that the diagnosis of FH should always be suspected in adults aged 20 and above with LDL-Cholesterol values greater than or equal to 190 mg/dL, as done in the present study due to the unavailability of genetic testing^27^.

In this study, clinical criteria of the DLCN were adopted. However, in addition to the clinical criteria we adopted, the presence of xanthomas (6 points) and corneal arch (4 points) are also part of the score's clinical diagnosis, which are evaluated by physical examination and positive family history (which varies between 1 and 2 points)^6^. In Brazil, in people with FH, the approximate prevalence of corneal arch is 28% and xanthomas 13%^28^. We do not have data from the physical examination and lipid profile of the family members. This possibly shows that the prevalence of probable cases or those that are classified as certain cases of FH may be underestimated^6^ in the present study. However, it should be noted that in the unavailability of genetic tests, the diagnosis must be based on LDL-cholesterol levels^29^. Furthermore, even in patients without genetic confirmation for FH, management must pay special attention to modifiable factors associated with LDL cholesterol^30^, which denotes the relevance of the results found here.

The high FH prevalence values found in this study may be influenced by not excluding secondary causes, such as hypothyroidism and nephrotic syndrome. The exclusion of secondary causes was not possible due to the lack of information in the PNS. Also the absence of genetic testing, although not mandatory in case of unavailability^24^, may have contributed to the inclusion of other dyslipidemias or metabolic diseases that lead to lipid alterations^7^. It is noteworthy that although we used the adapted DLCN criteria, individuals diagnosed with FH by criterion 2 are more likely to have severe hypercholesterolemia by behavioral and non-genetic causes, a possibility that can be corroborated by the higher prevalence of other metabolic alterations in this group when compared by criterion 1. However, considering that early diagnosis and treatment in FH reduce unfavorable clinical outcomes, it was decided to maintain more sensitive criteria, even losing in specificity.

Studies show that male and female can be equally affected by FH^1,31^. However, we identified a higher prevalence in women, as in Poland^32^, Catalonia^33^ and China^11^. There is evidence of gender disparities in FH treatment, with the disease having a different weight in women, with implications for treatment adherence^34^. Women are less likely to use statins and to discontinue therapy, and consequently may not reach recommended LDL-cholesterol levels^34^. The reasons are related to the challenges faced in the childbearing age regarding the choice of contraceptives and lipid-lowering therapy; discontinuation of treatment with lipid-lowering drugs because they are teratogenic during pregnancy, a phase in which LDL-Cholesterol levels are increased in FH; choice of therapy or restart during breastfeeding; and menopausal care in women with FH, in which LDL-Cholesterol levels are higher compared to men^34^. Another possible explanation is due to survival bias related to early and fatal manifestations of FH in men. However, the reasons for this discrepancy are uncertain^6^, explanations lack empirical and theoretical evidence; therefore, need to be further investigated.

Regarding race/color, the result of this investigation diverges from the Elsa-Brazil Study, in which FH affected more browns and blacks^6^, while a study in the United States identified higher prevalence in whites^17^. Another study in Brazil with laboratory data from the PNS identified lower prevalence of high LDL-Cholesterol in blacks and browns^35^. Possible justification is due to the adopted score^3,7,8^, which uses LDL-Cholesterol in its algorithm. There is no consensus in the literature regarding the genetic factors that allow predicting the highest prevalence of FH in certain ethnic-racial groups^36^. There is little information about FH in mixed-race populations^6^, making further research in the country necessary, especially due to miscegenation of Brazilians.

About the relationship of FH with age, a decrease in LDL-Cholesterol levels in FH is expected with advancing years^18^. However, in this study, the prevalence increased with age, as in studies in French^18^, Chinese^11^, Danish^26^ and Elsa-Brasil^6^. In the present investigation, as in others that adopted part of the score^6,11,17,18,26^, the prevalence was lower in young people^6,11,26^. However, this also occurred in research with more complete scoring algorithms, which included, in addition to LDL-Cholesterol levels and a history of premature CAD and stroke^6,11,17,18,26^, family history^6,11,18,26^ and/or genetic test^26^. Although this phenomenon may be attributable to not using all the score criteria, it implies that FH is underdiagnosed in young people, as it is a genetic condition^26^. On the other hand, not excluding secondary causes may have increased patients with severe metabolic disorders, and contributed to overestimating the prevalence of FH at older ages, when secondary causes are more prevalent^4^, as can be seen in the adults studied here between 45 and 59 years and 60 years or more. It is also important to mention that possible explanations may be due to lipid changes resulting from the aging process, which increase with advancing age. Aging mechanisms affect tissues and organs, resulting in changes in the hepatic endothelium, increased insulin resistance, androgen decrease in men and hormones in women resulting from menopause and post-climacteric^35^.

This study showed a negative association between higher education and the presence of FH. This finding is relevant as adults with FH who have low education are less likely to seek health care, adhere to treatment and advocate for tracking relatives^37,38^. Patients with FH with low education benefit, therefore, from educational interventions about the disease, constituting important tools for FH control, treatment and tracking among index cases^37,38^.

Another interesting association was the presence of FH in adults with worse self-rated health. A study with data from PNS showed a strong association between poor health self-assessment and dyslipidemias^39^. Health self-assessment is a predictor of morbidity and mortality and use of health services^39,40^. It expresses individuals’ social, psychological and biological dimensions and is related to a greater understanding of the diagnosis, symptoms, decreased functionality and risk of mortality^39,40^. Possible justifications for these findings can be explained by participants’ perception of the severity and risks of FH.

People with diabetes, hypertension and TC ≥ 310 mg/dL had higher prevalence of possible cases of FH, conditions that further increase the risk of CVD in the presence of FH^1,41^. In people with FH, diabetes increases the risk of CVD by 2.19-fold and hypertension confers the 1.4-fold greater risk of CVD^41^. Thus, it is important to emphasize to individuals with FH the relevance of diagnosis and adoption of treatment for these conditions^41^. Very increased TC values may be indicative of FH and excluding secondary dyslipidemias, these adults should be evaluated for the possibility of the disease^24^. A meta-analysis addressed the association between risk factors considered here and CVD in individuals with FH^42^. It should be noted that these associations in possible cases of FH in Brazilian adults can be confirmed in future studies. The literature is uncertain as to the association between LDL-cholesterol and CVD in FH, but record the small number of participants in studies that evaluated these associations, it may not be effective to show real effects of LDL-cholesterol differences in this population^42^. Furthermore, patients with very high LDL-cholesterol are, in general, treated more aggressively, introducing a confounding factor in the analyses^43^.

### Strengths and limitations

Among the contributions of this study, it is worth highlighting that the results can be useful for surveillance and guidance for policymakers and healthcare professionals in their clinical practice, as well as support strategies to address the burden of FH in Brazil. Additionally, due to the scarce data on FH in the country, it is essential to establish an early and systematic screening program in Brazil^9^. Screening increases the number of diagnoses, enabling early treatment and reduction of cardiovascular risk. Screening should be performed even in the absence of genetic testing, as it is considered a highly cost-effective strategy^7^. It is important to note that, in the absence of genetic testing, the diagnosis of FH should be based on LDL cholesterol levels and familial screening of confirmed cases^33^. Knowledge of the lipid profile assists in diagnosing a greater number of cases, considering the likelihood of other first-degree, second-degree, and third-degree relatives being carriers of the disease^44^. Screening increases the number of diagnoses, enabling early treatment and reduction of cardiovascular risk^7^.

It is also worth emphasizing that the identification of elevated LDL cholesterol levels, regardless of the diagnosis of FH, is relevant, as these individuals have a higher risk of morbidity and mortality from CVD and should be evaluated for the presence of secondary causes or indication of lipid-lowering treatment, according to cardiovascular risk assessment^45^. One fifth of Brazilian adults have high LDL-Cholesterol^35^, which constituted only in 2019, in Brazil, as the eighth cause of loss of disability-adjusted life years (DALYs), causing 2,363,140 million DALYs (3.62% of the total) and the sixth cause of deaths, causing 99,375 deaths (7.04% of the total)^46^.

Some limitations of this study should be considered. Firstly, the inability to establish a causal relationship, as the associations found may reflect lifestyle and FH treatment. Secondly, the absence of all scoring criteria and the inability to exclude secondary causes of dyslipidemia due to the lack of information in the PNS database, which may have led to an underestimation or overestimation of the prevalence. Thirdly, some results may be subject to survivorship bias and reverse causality and should be carefully evaluated. In this study, due to the lack of information in the PNS about genetic tests, clinical examination to identify xanthoma (and corneal arch under 45 years of age and family history of CVD and stroke, we were unable to reach the probable and definitive cases of FH of the scores DLCN. However, it is important to highlight that the information available about FH in Brazil is limited^5,8,9^ and there is little information about the distribution of the disease in the country^6–8^. To date, only one previous population study has been found with a sample composed of employees from Brazilian public universities^11^. Although we do not have exact and comprehensive data to define the disease based on the complete score, this study allowed us to estimate, for the first time, possible cases of FH and associated factors in a representative sample of Brazilians, using data from the broadest national health survey that is the PNS, bringing us closer to the country's reality and aligned with the efforts of the WHO^8^. Furthermore, in this study we also consider that according to the literature, the diagnosis of FH should be based on LDL cholesterol levels in the unavailability of genetic tests^29^.

## Conclusions

The frequency of possible cases of FH in adults in Brazil was 1:104. FH were negatively associated with being of black race/color and with higher education, and positively associated with regular health self-assessment, poor/very poor. The study showed that there was an increase in prevalence when estimating by more score criteria, showing that the inclusion of other score information can identify more individuals with FH. Diagnosis of possible cases of FH can help reduce the impact on cardiovascular morbidity and mortality in Brazilians by enabling early treatment, and are in line with WHO efforts to identify FH in low- and middle-income countries for CVD prevention.

### Supplementary Information

Supplementary Tables.

## Data Availability

The datasets generated during and/or analyzed during the current study are available in the Institute of Communication and Scientific and Technological Information in Health of Oswaldo Cruz Foundation (Icict/Fiocruz, in portuguese) repository, https://www.pns.icict.fiocruz.br/bases-de-dados/.
