# Supplementary material for: Prevalence and factors associated with possible cases of familial hypercholesterolemia in Brazilian adults: a cross-sectional study
Source: Sci Rep. 2023 Nov 22;13:20459. doi: 10.1038/s41598-023-47692-7 (PMC10665423; doi:10.1038/s41598-023-47692-7)
Supplement: Supplementary file 1 — Supplementary Tables. [file 41598_2023_47692_MOESM1_ESM.docx]

Supplementary Table 1– Questions from the Brazilian National Health Survey (PNS) questionnaire or indicators used to construct the variables, Brazil, 2014-2015

| **Variables** | **PNS questions or indicators used to construct the variables*** |
| --- | --- |
| Criterion 1 | LDL-Cholesterol^a^ - Collected value of LDL-Cholesterol in mg/dL. Answer options: LDL cholesterol levels (mg/dL): 155-189; 190-249; 250-329; ≥ 330. Variable code in PNS database Z033. |
| Criterion 2 | LDL-Cholesterol^a^ - Collected value of LDL-Cholesterol in mg/dL. Answer options: LDL cholesterol levels (mg/dL): 155-189; 190-249; 250-329; ≥ 330. Variable code in PNS database Z033.  Cardiovascular disease^b^ – Has a doctor ever given you the diagnosis of a heart disease, such as a heart attack, angina, heart failure or something else? Answer options: Yes; No. Variable code in PNS database Q63.  Stroke^b^ – Has a doctor ever given you the diagnosis of a cerebrovascular accident (CVA) or stroke? Answer options: Yes; No. Variable code in PNS database Q68.  Questions that were used to construct the variable cardiovascular disease and premature stroke used in criterion 2. The positive report of these diseases was considered premature in men under 55 years and women under 60 years ^b^. |
| Overweight or obesity | Final weight in kilograms^c^. Variable code in PNS database Z004.  Final weight in kilograms ^c^. Variable code in PNS database Z005.  Indicators used to construct the overweight and obesity variable using the body mass index (BMI) formula. The following answer options were considered: normal/underweight (BMI < 25 kg/m^2^), overweight (BMI between 25 to 29 kg/m^2^) and obesity (BMI ≥ 30 kg/m^2^) ^c^. |
| Sufficient physical activity in free time | In the last three months, have you practiced any type of physical exercise or sport? Answer options: Yes; No. Variable code in PNS database Q34 ^b^.  How many days a week do you usually practice physical exercise or sport? Response options: Number of days; Never or less than once a week. Variable code in PNS database Q35 ^b^.  What physical exercise or sport do you practice most often? Answer options: Walk (not for work); aerobics/spinning/step/jump; Martial Arts and Fighting; Treadmill walk; Hydrogymnastics; Bicycle/exercise bicycle; Volleyball; Sneakers; running/jogging; General gymnastics/localized/pilates/stretching/yoga; Soccer; treadmill running; Swimming; Basketball; Dance (with the aim of practice physical activity); Other (Specify). Variable code in PNS database Q36 ^b^.  In general, on the day you practice exercise or sport, how long does this activity last? Response options: hours and minutes. Variable code in PNS database Q37 ^b^.  Questions that were used to construct the variable Sufficient physical activity in free time: It was considered practice of 150 minutes per week of PA of light or moderate intensity, or at least 75 minutes per week of vigorous intensity, it was considered active, regardless of the number of days of practice per week ^b^. |
| Consumption of red meat with fat | When you eat red meat, do you usually remove visible excess fat or eat with fat? Answer options: Remove visible excess fat; Eat the fat. Variable code in PNS database P12 ^b^. |
| Binge drinking | How many days a week do you usually drink any alcoholic beverage? Response options: Days; Never or less than once a week. Variable code in PNS database P28 ^b^.  In general, on the day you drink, how many doses of alcohol do you consume? (1 dose of alcoholic beverage is equivalent to 1 can of beer, 1 glass of wine or 1 dose of cachaça, whiskey or any other distilled alcoholic beverage). Answer options: Number of doses. Variable code in PNS database P29 ^b^.  Questions that were used to construct the variable Binge drinking. A minimum consumption of 4 doses for women and 5 doses for men in a single occasion was considered ^b^. |
| Smoking | Do you currently smoke any tobacco products? As answer options, the following statements were adopted: Answer options: Yes, daily; Yes, less than daily; I currently don't smoke. Variable code in PNS P50 database ^b^. |
| Health self-assessment | In general, how do you rate your health? Answer options: Very good; Good; Regular; Bad; Too bad. Variable code in PNS database N1 ^b^. |
| Diabetes | Glycated hemoglobin^a^ – Collected value of Glycated Hemoglobin in percentage (%). Response options: greater than or equal to 6.5%. Variable code in PNS database Z034.  Self-reported diagnosis of diabetes^b^ - Has a doctor ever given you a diagnosis of diabetes? Answer options: Yes; Only during pregnancy; No. Variable code in PNS Q30 database. |
| Hypertension | Blood Pressure^a^ – Measured blood pressure value in mmHg. Variable code in PNS database Z034. Response options: ≥ 140/90 mmHg. Variable code in the PNS database W00407 (systolic blood pressure) and W00408 (diastolic blood pressure).  Self-reported diagnosis of Hypertension^d^ - A doctor has already given you the diagnosis of high blood pressure (high blood pressure). Answer options Answer options: Yes; Only during pregnancy; No. Variable code in PNS database Q02; In the last two weeks, have you taken medication because of high blood pressure (high blood pressure)? Answer options: Yes; Not; Variable code in PNS Q06 database. |
| Total cholesterol (TC) ≥ 310 mg/dL | TG collected value in mg/dL^a^. Response options: ≥ 310 mg/dL. Variable code in PNS database Z031. |
| HDL ≤ 40 mg/dL | Collected value of HDL-Cholesterol in mg/dL^a^. Response options: ≤ 40 mg/dL. Variable code in PNS database Z032. |
| Treatment report | Report of antihypertensive treatment - In the last two weeks, have you taken medication because of high blood pressure (high blood pressure)? Answer options: Yes; Not; Variable code in PNS Q06 database ^b^.  **Lipid-lowering treatment report - Has a doctor or other healthcare professional given you any of the following recommendations because of high cholesterol? Taking Medications: Answer Options: Yes; No. Variable code in PNS database Q06204 ^b^. |

*This study used the PNS laboratory database and the survey questionnaire modules for the selected adult resident, with these resources being available on the PNS website (link: https://www.pns.icict.fiocruz.br/). Questions from the PNS questionnaire of the modules were used: general identification of the selected participant; P — on lifestyles (risk and protective factors for CNCDs, such as use of tobacco, alcohol, food, physical activity); Q — about diseases chronicles; N — about perception of health status; and W — on anthropometry. a) Parameters dosed by laboratory tests of the PNS; b) Self-reported measures. Questions from the PNS questionnaire were used modules P (lifestyles), Q (chronic non-communicable diseases) and N (Health self-assessment) c) The body mass index (BMI), which was calculated from measurements of weight and height measured in the PNS. Questions from the PNS questionnaire were usedof the W module (anthropometry). d) The self-reported diagnosis for arterial hypertension set out in Module Q was used of the PNS questionnaire and blood pressure measurements taken in the PNS.

Supplementary Table 2 - Prevalence of LDL-Cholesterol and 95%CI in adults by range Dutch Lipid Clinic Network (DLNC) according to sociodemographic characteristics, Brazilian National Health Survey, Brazil, 2014-2015

|  |  | **< 155 mg/dL** | | | **155-189 mg/dL** | | | **190-249 mg/dL** | | | | **250-329 mg/dL** | | | |  |  |
| --- | --- | --- | --- | --- | --- | --- | --- | --- | --- | --- | --- | --- | --- | --- | --- | --- | --- |
|  |  | **(0 points)*** | | | **(1 point)*** | | | **(3 points)**** | | | | **(5 points)**** | | | |  |  |
|  |  | **n=8,035** | | | **n=420** | | | **n=63** | | | | **n=3** | | | |  |  |
| **Variables** | **n** | **%** | | **95%CI** | **%** | **95%CI** | | **%** | | **95%CI** | | **%** | | **95%CI** | | |  |
| **Total** | 8,521 | 94.47 | | 93.82-95.06 | 4.84 | 4.29-5.45 | | 0.64 | | 0.46-0.89 | | 0.044 | | 0.01-0.17 | | |  |
| **Sex** | 8,521 |  | |  |  |  | |  | |  | |  | |  | | |  |
| Male |  | 95.71 | | 94.78-96.48 | 3.83 | 3.10-4.73 | | 0.41 | | 0.24-0.72 | | 0.04 | | 0.01-0.25 | | |  |
| Female |  | 93.37 | | 92.42-94.20 | 5.74 | 4.97-6.62 | | 0.85 | | 0.57-1.27 | | 0.05 | | 0.01-0.33 | | |  |
| **Age** | 8,521 |  | |  |  |  | |  | |  | |  | |  | | |  |
| 20 to 29 |  | 97.81 | | 96.48-98.65 | 1.72 | 1.01-2.90 | | 0.38 | | 0.10-1.41 | | 0.09 | | 0.01-0.64 | | |  |
| 30 to 44 |  | 96.33 | | 95.29-97.15 | 3.35 | 2.55-4.40 | | 0.31 | | 0.17-0.55 | | 0.01 | | 0.001-0.05 | | |  |
| 45 to 59 |  | 91.69 | | 90.10-93.04 | 7.39 | 6.12-8.91 | | 0.83 | | 0.47-1.48 | | 0.09 | | 0.01-0.65 | | |  |
| 60 years and older |  | 92.13 | | 90.63-93.42 | 6.74 | 5.56-8.15 | | 1.13 | | 0.69-1.85 | | 0.00 | | 0.00-0.00 | | |  |
| **Education** | 8,521 |  | |  |  |  | |  | |  | |  | |  | | |  |
| Illiterate/Incomplete elementary school |  | 93.63 | | 92.61-94.52 | 5.29 | 4.48-6.23 | | 0.97 | | 0.66-1.43 | | 0.11 | | 0.03-0.44 | | |  |
| Complete elementary school/incomplete high school |  | 95.26 | | 93.63-96.48 | 4.29 | 3.11-5.89 | | 0.44 | | 0.21-0.94 | | 0.01 | | 0.002-0.09 | | |  |
| Complete high school and more |  | 94.92 | | 93.86-95.80 | 4.65 | 3.81-5.66 | | 0.43 | | 0.21-0.89 | | 0.00 | | 0.00-0.00 | | |  |
| **Race/skin color** | 8,521 |  | |  |  |  | |  | |  | |  | |  | | |  |
| White and others |  | 93.94 | | 92.86-94.86 | 5.15 | 4.29-6.16 | | 0.82 | | 0.53-1.29 | | 0.09 | | 0.02-0.34 | | |  |
| Black |  | 96.30 | | 94.32-97.6 | 3.65 | 2.35-5.63 | | 0.05 | | 0.007-0.37 | | 0.00 | | 0.00-0.00 | | |  |
| Brown |  | 94.70 | | 93.82-95.46 | 4.74 | 4.02-5.59 | | 0.56 | | 0.36-0.88 | | 0.00 | | 0.00-0.00 | | |  |
| **Region** | 8,521 |  | |  |  |  | |  | |  | |  | |  | | |  |
| North |  | 95.75 | | 94.74-96.57 | 3.57 | 2.82-4.52 | | 0.66 | | 0.38-1.12 | | 0.03 | | 0.004-0.21 | | |  |
| Northeast |  | 94.39 | | 93.51-95.16 | 4.97 | 4.24-5.81 | | 0.64 | | 0.42-0.97 | | 0.00 | | 0.00-0.00 | | |  |
| Southeast |  | 94.31 | | 93.02-95.38 | 5.00 | 4.01-6.23 | | 0.63 | | 0.33-1.21 | | 0.05 | | 0.01-0.39 | | |  |
| South |  | 94.27 | | 92.62-95.57 | 5.13 | 3.90-6.72 | | 0.48 | | 0.21-1.09 | | 0.12 | | 0.02-0.83 | | |  |
| Center-West |  | 94.95 | 93.38-96.17 | | 4.00 | | 2.93-5.44 | | 1.05 | | 0.57-1.92 | | 0.00 | | 0.00-0.00 | | |

95%CI: 95% confidence interval. The category of LDL-Cholesterol levels ≥ 330 mg / dL was not presented due to the lack of cases. *Scores between 0 and 1 points represent unlikely cases of familial hypercholesterolemia according to the Dutch Lipid Clinic Network (DLNC) LDL cholesterol ranges. LDL-cholesterol values below 155 mg/dL correspond to 0 points on the score (referring to unlikely cases of familial hypercholesterolemia) and LDL-cholesterol values between 155-189 mg/dL correspond to 1 point on the mg/dL score (refers to unlikely cases of familial hypercholesterolemia). **Scores between 3 and 5 points represent possible cases of familial hypercholesterolemia according to the Dutch Lipid Clinic Network (DLNC) LDL cholesterol ranges. LDL cholesterol. Values between 190-249 mg/dL correspond to 3 points in the score (referring to possible cases of familial hypercholesterolemia) and LDL-cholesterol values between 250-329 mg/dL correspond to 5 points in the mg/dL score (referring to possible cases of familial hypercholesterolemia).

Supplementary Table 3 – Prevalence of possible cases of FH in adults by criteria according to additional risk factors for cardiovascular diseases, altered laboratory tests and treatment report, Brazilian National Health Survey, Brazil, 2014-2015

|  |  | **Possible cases of FH by Criterion 1** | | | **Possible cases of FH by Criterion 2** | | |
| --- | --- | --- | --- | --- | --- | --- | --- |
|  |  | **(3-5 points)** | | | **(3-5 points)** | | |
|  |  | **(n=66)** | | | **(n=87)** | | |
| **Variables** | **n *** | **%** | **95%CI** | **p** | **%** | **95%CI** | **p** |
| **Hypertension** | 8,146 |  |  |  |  |  | 0.002 |
| Yes |  | 1.04 | 0.64-1.68 | 0.098 | 1.75 | 1.16-2.63 |  |
| No |  | 0.61 | 0.39-0.92 |  | 0.75 | 0.52-1.10 |  |
| **Diabetes** | 8,293 |  |  |  |  |  | 0.02 |
| Yes |  | 1.33 | 0.72-2.43 | 0.031 | 1.85 | 1.07-3.18 |  |
| No |  | 0.62 | 0.43-0.89 |  | 0.87 | 0.63-1.20 |  |
| **Total cholesterol ≥310(mg/dL)** | 8,520 |  |  |  |  |  | <0.001 |
| Yes |  | 53.95 | 28.59-77.42 | <0.001 | 53.95 | 28.59-77.42 |  |
| No |  | 0.49 | 0.32-0.73 |  | 0.76 | 0.55-1.05 |  |
| **HDL-Cholesterol ≤40(mg/dL)** | 8,506 |  |  |  |  |  | 0.91 |
| Yes |  | 0.88 | 0.52-1.47 | 0.232 | 0.98 | 0.61-1.59 |  |
| No |  | 0.59 | 0.39-0.87 |  | 0.95 | 0.68-1.31 |  |
| **Antihypertensive treatment** | 2,023 |  |  |  |  |  | 0.363 |
| Yes |  | 0.86 | 0.73-4.88 | 0.140 | 1.62 | 0.99-2.62 |  |
| No |  | 1.92 | 3.81-10.59 |  | 2.47 | 1.13-5.31 |  |
| **Lipid-lowering treatment** | 1,212 |  |  |  |  |  | 0.647 |
| Yes |  | 2.75 | 1.73-4.36 | 0.904 | 3.29 | 2.11-5.09 |  |
| No |  | 2.58 | 0.99-6.55 |  | 2.58 | 0.99-6.55 |  |

*Missing data not presented. The category not having FH was used to calculate the prevalence, but it is not shown in the table. FH: familial hypercholesterolemia. 95%CI: 95% confidence interval.

Supplementary Table 4 – Prevalence of possible cases of FH in adults by criterion 1 according to selected variables, Brazilian National Health Survey, Brazil, 2014-2015

|  | **Possible cases of FH by Criterion 1** | | | |
| --- | --- | --- | --- | --- |
|  | **(3-5 points)** | | | |
|  | **(n=66)** | | | |
| **Variables** | **n*** | **%** | **95%CI** | **p** |
| **Total** | 8,521 | 0.69 | 0.50-0.94 |  |
| **Sex** | 8,521 |  |  |  |
| Male |  | 0.46 | 0.27-0.78 | 0.043 |
| Female |  | 0.89 | 0.60-1.32 |  |
| **Age** | 8,521 |  |  |  |
| 20 to 29 |  | 0.47 | 0.15-1.45 | 0.044 |
| 30 to 44 |  | 0.32 | 0.18-0.56 |  |
| 45 to 59 |  | 0.92 | 0.53-1.60 |  |
| 60 years and older |  | 1.13 | 0.70-1.85 |  |
| **Race/skin color** | 8,521 |  |  |  |
| White and others |  | 0.91 | 0.60-1.40 | 0.012 |
| Black |  | 0.052 | 0.007-0.37 |  |
| Brown |  | 0.56 | 0.36-0.88 |  |
| **Region** | 8,521 |  |  |  |
| North |  | 0.68 | 0.40-1.15 | 0.771 |
| Northeast |  | 0.64 | 0.42-0.97 |  |
| Southeast |  | 0.69 | 0.37-1.27 |  |
| South |  | 0.60 | 0.28-1.28 |  |
| Center-West |  | 1.05 | 0.57-1.92 |  |
| **Education** | 8,521 |  |  |  |
| Illiterate/Incomplete elementary school |  | 1.08 | 0.74-1.57 | 0.019 |
| Complete elementary school/incomplete high school |  | 0.46 | 0.22-0.95 |  |
| Complete high school and more |  | 0.43 | 0.21-0.88 |  |
| **Body Mass Index** | 8,429 |  |  |  |
| Low/Normal |  | 0.45 | 0.26-0.79 | 0.120 |
| Overweight |  | 0.98 | 0.62-1.54 |  |
| Obesity |  | 0.63 | 0.30-1.38 |  |
| **Physical activity** | 8,511 |  |  |  |
| No |  | 0.79 | 0.56-1.11 | 0.035 |
| Yes |  | 0.33 | 0.15-.071 |  |
| **Consumption of red meat with fat** | 8,041 |  |  |  |
| No |  | 0.51 | 0.25-1.03 | 0.242 |
| Yes |  | 0.81 | 0.56-1.16 |  |
| **Binge drinking** | 8,521 |  |  |  |
| No |  | 0.60 | 0.24-1.50 | 0.601 |
| Yes |  | 0.69 | 0.50-0.97 |  |
| **Smoking** | 8,514 |  |  |  |
| No |  | 1.17 | 0.55-2.47 | 0.107 |
| Yes |  | 0.60 | 0.43-0.85 |  |
| **Health self-assessment** | 8,514 |  |  |  |
| Very good/good |  | 0.53 | 0.33-0.845 | 0.111 |
| Regular |  | 0.93 | 0.57-1.51 |  |
| Very poor/poor |  | 1.24 | 0.50-3.02 |  |

*Missing data not presented. The category not having FH was used to calculate the prevalence, but it is not shown in the table. FH: familial hypercholesterolemia. 95%CI: 95% confidence interval.
